# Power Dynamics in a Triad Relationship: Advance Care Planning for Older Adults with Dementia in China

**DOI:** 10.1093/geroni/igaa057.3324

**Published:** 2020-12-16

**Authors:** Yifan Lou, Jinyu Liu, Bei Wu

**Affiliations:** 1 Columbia University, New York, New York, United States; 2 New York University, New York, New York, United States

## Abstract

Objective: Primary family caregiver (CG), other family members (FM), and medical professionals (MP) play important roles in medical decision-making for older adults with dementia, who often have lost the capacity to make decisions on their own. Power dynamics within the CG-FM-MP triad relationship determine the process and outcome of the decision-making. Guided by Rahl’s relational power model, this study is among the first to understand the experiences of advance care planning among Chinese. Method: This study includes a total of 25 primary CGs or FMs and 5 MPs from 3 neurology departments. Hybrid grounded theory method was used to analyze the preliminary data we had so far. Based on the dimensions of power, we analyzed the power base, means, and scope of each agent in each interview to determine the power comparability. Results: Three types of triadic power relations were categorized: 1) shared-power with shared-decision, in which three agents shared the power of decision-making and CG as the lawful decision-maker makes the final decisions; 2) balanced-power with reversed-patriarchal decisions, in which FM’s power is over both CG and MP and become the actual decision-maker; and 3) unbalanced power with conflicting decisions, in which neither CG and FM has absolute power over each other and MP becomes the actual decision-maker implicitly. Conclusion: The study provides a framework for researchers and practitioners to understand the ACP process for Chinese older adults, which helps develop intervention strategies to improve surrogates’ ACP knowledge and reduce potential conflicts during the stressful process for the population.

